# Patterns and Predictors of Off-Label Drug Prescribing in Psychiatric Practice: A Qualitative Study

**DOI:** 10.3390/pharmacy9040203

**Published:** 2021-12-20

**Authors:** Sadia Shakeel, Shagufta Nesar, Hina Rehman, Khizra Jamil, Imran Ahsan Mallick, Muhammad Shahid Mustafa, Mudassir Anwar, Shazia Jamshed

**Affiliations:** 1 Department of Pharmacy Practice, Faculty of Pharmaceutical Sciences, Dow College of Pharmacy, Dow University of Health Sciences, Karachi 74200, Pakistan; sadia.shakeel@duhs.edu.pk (S.S.); imran.ahsan@duhs.edu.pk (I.A.M.); 2 Jinnah College of Pharmacy, Sohail University, Karachi 74000, Pakistan; Shaguftausmani@sohailuniversity.edu.pk; 3 Department of Pharmacy Practice, Institute of Pharmaceutical Sciences, Jinnah Sind Medical University, Karachi 75510, Pakistan; hina.rehman@jsmu.edu.pk; 4 Department of Psychology, University of Karachi, Karachi 75270, Pakistan; khizrajamil@hotmail.com; 5 Diplomat American Board of Psychiatry and Neurology, Neuro Care Clinic, Karachi 75100, Pakistan; smustafa63@hotmail.com; 6 School of Pharmacy, University of Otago, Dunedin 9054, New Zealand; mudassir.anwar@otago.ac.nz; 7 Department of Clinical Pharmacy and Practice, Faculty of Pharmacy, Universiti Sultan Zainal Abidin, Kuala Terengganu 21300, Malaysia

**Keywords:** attitude, knowledge, psychiatrists, qualitative study, off-label drug prescribing, Pakistan

## Abstract

Off-label drug prescribing (OLDP) must be based on strong scientific evidence to make sure that patients get the optimum therapeutic outcomes. Adherence to the prerequisites is determined by the physicians’ attitude and knowledge. In this context, the present study was conducted with the goal of investigating psychiatrists’ perceptions of the use of OLDP in their clinical practice. A total of 14 psychiatrists were interviewed using a semi-structured interview guide. Thematic content analysis was performed. Data saturation was achieved at the 12th interview. Six major themes and fifteen subthemes emerged from qualitative interviews. Among the major themes were knowledge and concepts about the off-label drugs, attitude and current practice of prescribing off-label drugs, and rationale of prescribing and suggestions for reducing the use of off-label drugs. Almost all of the respondents interviewed provided detailed comments concerning the OLDP concept, depicted an optimistic approach and deemed that OLDP is quite common in psychiatry. Off-label usage of benzodiazepines such as clonazepam, diazepam and lorazepam in mania, depression, and obsessive–compulsive disorder were commonly reported. It was observed that the majority of the respondents did not inform the patients before prescribing off-label drugs. The present findings revealed that respondents had awareness; however, they depicted diverse attitudes towards prescribing off-label drugs. Further education and sensitization in regions with impoverished knowledge would certainly assist in preventing the risks associated with the use of OLDP.

## 1. Introduction

Many medications prescribed to patients are not approved for the specific diagnosis, patient age, or dose, and their usage in these instances is referred to as “off-label” or “unlicensed” prescription, or the “use of licensed pharmaceuticals for an unapproved indication” [1]. Medication prescription for off-label reasons is becoming more prevalent; recent occurrences depicted such prescribing as one of the pillars of evolving clinical therapy. Off-label drug prescription (OLDP), by doctors across all medical specialties has increased significantly in preceding decades because it allows them to employ novel treatment alternatives based on the most recent data [2].

The scarcity of controlled clinical trials in psychiatric patients is the key factor for the common practice of OLDP [3]. Many physicians consider that OLDP has a significant role in therapeutic practice, representing the optimum way to utilize that treatment, and it is frequently required when treating certain patients, such as those whose symptoms have proved resistant to a variety of therapeutic options [4]. Peer-reviewed publication publishes information regarding novel applications of medication which allows the physicians to use innovative therapeutic choices based on the most recent research [5]. However, the inappropriate use of OLDP could generate serious concerns regarding efficacy, safety, and adverse drug reactions (ADRs) as observed in previous research [6,7].

The extent of OLDP in psychiatric practice is unknown in Pakistan, although research studies indicate that it is widespread across all psychiatric domains [8]. The use of psychotropic medication for an unapproved reason does not always suggest a safety risk, and there are numerous cases when the usage is uncontroversial and likely beneficial to the patient [9]. Sometimes it might be useful to consider a spectrum of approved psychotropic medication usage in unapproved applications, with specific prescribing considered as “near-label” (for instance, the antidepressant fluoxetine’s use as a maintenance treatment in a recurrent depression patient) [10,11,12]. Even when a drug has gone through clinical trials and is proven to be effective and safe, there remains a delay in gaining regulatory clearance for a new indication. At times the OLDP is required, as >80% of the DSM-IV psychiatric diagnoses have no Food and Drug Administration (FDA)-approved medicine [13]. However in the preceding two decades, clinical trials have increased probing for new therapeutic clues for previously recognized psychiatric drugs, e.g., gabapentin in insomnia and anxiety disorders and duloxetine in neuropathic pain [13].

Prior research has sought to acquire an understanding of healthcare professionals’ perspectives and experiences with the use of OLDP [14,15]. Healthcare workers are shown to have a moderate level of knowledge of this issue, which they gained during their professional experience and growth, as well as their undergraduate and post-graduate education and training [6]. In Pakistan, being a developing country, the economic burden of OLDP could further deteriorate the situation and therefore it is imperative to study this issue in a local context. The present study would build on previous studies by investigating knowledge and attitudes concerning the use of OLDP in the psychiatric field. The significance of this work arises from the fact that earlier work dealing with OLDP has been carried out in the United States, the United Kingdom, and other nations across the world, and there is a scarcity of data from Pakistan [2,3,15,16]. Given the scarcity of studies on prescribing trends of healthcare professionals in Pakistan regarding the use of OLDP, the current qualitative method was designed to explore knowledge, attitude, and practices and to fill the gaps in the literature regarding OLDP in psychiatric practice.

## 2. Materials and Methods

### 2.1. Study Design and Setting

The current study was an explorative and qualitative study design that was based on face-to-face, in-depth interviews with psychiatrists to determine their views about OLDP. The qualitative methodology was chosen for a variety of reasons, including its flexibility and ability to provide in-depth insight, which aids in a deeper understanding of respondents’ awareness of the topic [17]. It explores the experiences, underlying beliefs, opinions or preferences of a targeted population, or to better comprehend a certain behavior and what influences it. Furthermore, the qualitative research provides comprehensive knowledge into a problem which could generate hypotheses for quantitative research [18,19].

The psychiatrists were recruited from two private and two public sector tertiary care hospitals in Karachi and were asked to participate in the study. Karachi is Pakistan’s biggest city and the world’s twelfth-largest city. It is the capital of Pakistan’s Sindh province. It has a population of about 16 million people and serves as the commercial hub of the country [20].

### 2.2. Development of Interview Guide

A semi-structured guide was developed based on a comprehensive review of the literature and the practices of OLDP reported previously [14,21,22,23,24,25]. The guide was intended to explore the respondent’s knowledge about OLDP, their concerns about its safety and efficacy, the probability of ADRs and their current practices of OLDP. The respondents’ suggestions for reducing off-label use and their practice of informing patients about OLDP were also documented. Two researchers with relevant backgrounds from the Dow University of Health Sciences reviewed and validated the guide. The reliability was assured by face-to-face interviews with the respondents. The semi-structured interview guide was pilot tested with four psychiatrists (who were not included in the final study), with researchers going over the questions with them to check for their understanding of vocabulary, for the relevance and importance of questions, and for their ability to reply the questions. The respondents were able to comprehend the terms that were used in the interview guide, questions were meaningful, and they thought the questions were relevant to their experience and/or were effective in tapping what they knew. Based on their responses and feedback, two questions were rephrased, and one question was omitted. The final interview guide was comprised of two segments; the first part focused on the demographic information of the respondents, whereas the second part included ten questions that assessed respondents’ knowledge and practices regarding OLDP in in psychiatric practice in Pakistan.

### 2.3. Study Sampling

Sampling technique used was a non-probability sampling. The needed sample size for the qualitative study was calculated till no innovative data emerged; the saturation point [26]. Saturation is defined as the development of theoretical components of the investigation. It is determined by the generation of rich information during the investigation process by considering the scope and replication. The scope of data in qualitative research refers to the comprehensiveness and depth of the issue studied, replication refers to data that has mutual but important characteristics from different respondents [27].

### 2.4. Inclusion Criteria

The respondents in the present study were registered psychiatrists from Pakistan Psychiatric Society and were rendering their services in tertiary care hospitals in Karachi. They were selected purposively and their suitability as per time and place availability was a consideration for their inclusion in this study. The psychiatrists not willing to participate were excluded. All the psychiatrists volunteered to take part in this study, and no monetary compensation was provided.

### 2.5. Data Collection

Prior to participating in the interview, each respondent was given an explanatory statement outlining the study’s goals, and written consent was acquired from them. They were informed about their right to withdraw from the study at any time. The identities of the respondents were kept confidential so that they had the opportunity to freely express their views. The interview was conducted by researchers who were trained on the relevant skills and had prior experience of conducting qualitative interviews.

The interviews were held at a time and location convenient for the respondents, mostly at the respondents’ offices or in a quiet room located on the ward, to allow for confidentiality and privacy. The interviews were conducted in English since respondents were fluent in the language and it is the country’s medium of education. Each interview lasted 30–45 min. To obtain the essential information, the questions were asked from the responders, and further field notes were gathered. The researcher audio-recorded and transcribed the interviews verbatim. Data saturation was reached after the twelfth interview, though two further interviews were performed to check if any different themes emerged.

### 2.6. Data Analysis

The data were manually evaluated by reading and rereading the interviews, and the study team used an inductive and flexible method. The assigned co-authors examined the interview transcripts to ensure that the generated codes and themes accurately reflected the contents of the interviews, and a mutual agreement was reached among all assigned study group members. A thematic content analysis was performed. Table 1 provides an overview of the various steps of analysis. The COREQ (consolidated criteria for reporting qualitative research) checklist for reporting qualitative studies aided the reporting of the methods and results [28].

### 2.7. Ethical Considerations

The study was conducted as per the recommendations of the Declaration of Helsinki and approval was obtained from the Ethical Review Committee of Sohail University, Karachi, Pakistan with the protocol # 000125/21. Written consent was obtained from the respondents before the study.

## 3. Results

### 3.1. Demographic Characteristics of the Respondents

Initially, 16 psychiatrists provided their consent to participate in the study; however, two of them refused at the time of the interview because of their busy schedules. A total of 14 psychiatrists aged between 30 to 60 years were interviewed. Table 2 illustrates the demographic characteristics of respondents. Among the respondents, eight were males and six were females. Half of the respondents (*n* = 7) were in between the age of 40–50 years and have an experience of 1–5 years. The majority of the respondents (*n* = 9) were working in private healthcare settings.

### 3.2. Thematic Analysis of the Content

Six primary topics emerged from the thematic content analysis of the interview. Table 3 below summarizes these themes and subthemes.

#### 3.2.1. Theme 1: Knowledge and Concepts about the Off-Label Drugs

##### Subthemes 1: Knowledge about the Definition

Almost all of the respondents interviewed provided adequate information concerning the OLDP. This demonstrates that nearly all respondents were familiar with the basic concept of OLDP.

*“Off label drugs are something that are not authorized to use in other than prescribed condition.”*

P-1

*“Off label medicine is defined as unapproved medicine from food and drug authority. These medicines are made for some other condition but are utilized for some other purpose. For example; using antidepressant for weight loss.”*

P-3

*“When treating a patient sometimes doctors prescribe medicine which are not directly related to illness or disease. Also, that medicine is not scientifically approved or validated by drug authority. This is off-label medicine…”*

P-14

*“Unapproved medication for any disorder is off label. Drug authority usually approves medicine for specific purpose. Those medicines if used for other than suggested purpose or group of patients are off-label medicines.”*

P-11

##### Subthemes 2: Knowledge about the Examples of Widely Practiced OLDP

The majority of the respondents deemed that OLDP is quite common in psychiatric practice.

*“In my opinion… the most common reason for off-label prescription are inappropriate indication. Like …Benzodiazepines are commonly used in schizophrenia.”*

P-5

*“Tricyclic Antidepressants (TCA) like amitriptyline had the highest prevalence of off-label indications for pain, migraine etc…”*

P-9

*“Benzodiazepines such as clonazepam, lorazepam, and diazepam… I believe it will be a surprise for us to know that many of the medications we use are off-label, since when we use certain medications so often, we forget that it is off-label and we consider that... it’s alright... it’s safe.”*

P-11

*“There are so many examples as Sodium valproate is widely used off-label in psychiatric patients with schizophrenia, particularly in younger patients.”*

P-4

##### Subthemes 3: Knowledge about the Policies and Guidelines

Despite having a basic understanding of the OLDP concept, some of the respondents were not well informed about OLDP policies and guidelines. A couple of the respondents stated that there are no guidelines or regulations of off label use in their working hospital.

*“Yes, only FDA-approved medicines are allowed to be prescribed…”*

P-3

*“It is a very common practice in Pakistan because there are no regulations in place to keep a check over the prescribing and dispensing pattern of medicines to patients.”*

P-7

*“Off-label usage in the absence of evidence base is associated with the risk to patients. Therefore, when prescribing off-label medicines, we employ databases for scientific literature searches and evidence-based treatment guidelines to reduce the risk of poor outcomes associated with off-label usage.”*

P-9

#### 3.2.2. Theme 2: Positive Attitude about OLDP

On inquiring about the attitude towards OLDP the majority of respondents (*n* = 8) depicted an optimistic approach. They opined that in the current age of global medical revolution, this prescribing practice may be more effective than limiting practice to indications mentioned in the drug labeling system.

##### Subthemes 1: Optimistic Approach about Safety, Efficacy, and Quality

*“Off-label prescription isn’t always a negative thing. It can be useful, especially when patients have exhausted all other treatment choices, like in the case of rare disorders.”*

P-2

*“Drugs are usually safe, there may be some concerns regarding efficacy but usually they are good to go with.”*

P-13

##### Subthemes 2: Willingness to Prescribe Off-Label Drugs

*“I would definitely prescribe off-label drug … if my patient would not be having certain FDA-approved medication option.”*

P-1

*“First, I’ll discuss with my colleagues... I’ll ask for their feedback... I’ll consult with the clinical pharmacists... yeah… Then, if it can be continued, I will provide…”*

P-14

Three respondents showed the neutral approach.

*“The key challenge is how to control off-label medication usage without limiting therapeutic innovation. I would emphasis on evidence-based prescribing… as well as stringent guidelines for off-label medication usage. If we will completely eliminate the off-label medications prescribing then it might impede patients’ early access to new medicines.”*

P-13

*“In my point of view, the off-label prescription of medicines is neither right nor wrong…. It is determined by the doctor’s grounds for prescribing off-label.”*

P-9

*“Not completely safe but it is better to give some treatment than no treatment… When something does not work doctors have to use them.”*

P-10

#### 3.2.3. Theme 3: Negative Attitude about OLDP

Three respondents demonstrated negative attitude towards the use of OLDP.

##### Subthemes 1: Likelihood of ADRs

*“Off-label medication usage can cause negative implications as these medicines have not undergone the testing needed by the FDA.”*

P-8

*“I don’t think so they are safe because all medicine have side effects and especially if some medication is used as off label, chances are double. Therefore, patients are more likely to have an adverse event, such as a medication reaction, or allergic response if they are administered off-label medicines.”*

P-4

*“I don’t prescribe them… but I have concerns if a patient is already using it because it is not safe and its effectiveness is not determined. Nothing is for sure when we give off-labels.”*

P-12

##### Subthemes 2: Legal Vulnerability When Using Off-Label Drugs

Some respondents deemed that for limiting the liability, we should only prescribe medications for the indications that we believe are most suitable for the patient and those only which are based on the most credible evidence available.

*“Physicians can unnecessarily involve themselves in legal proceedings if there is an adverse reaction to a drug administered for off-label usage.”*

P-6

*“If it comes to legal liability… I doubt it would stand since you are not meant to use it. Because that specific drug is only licensed for some specific purpose, and it shows that you are encouraging unauthorized use... in that scenario, I would say it’s illegal…”*

P-4

#### 3.2.4. Theme 4: Current Practice of Prescribing Off-Label Drugs

The respondents consider the OLDP as a common practice in Pakistan.

*“Often we do not realize that certain unapproved use of antidepressants is not based on evidence, especially if the wider community of physicians prescribes antidepressants for these unapproved uses so frequently, it seems to be the norm.”*

P-5

*“I have lived in Florida and the ratio is more than one in five outpatient prescriptions written in the U.S. are for off-label medications. In my opinion, in Pakistan, the ratio is higher than from other developed countries.”*

P-10

*“In other countries there are proper procedure of visiting a doctor and getting prescribed medicine. In Pakistan even those who are not specialized in mental condition prescribe psychiatric medicine. So, it’s extremely common practice. I will say everyone is experimenting on patients.”*

P-3

##### Subthemes 1: Category of Off-Label Drug Most Commonly Used

The respondents in the present study revealed a higher use of benzodiazepines as OLDP. Off-label usage of benzodiazepines such as clonazepam, diazepam, and lorazepam in mania, depression, and obsessive–compulsive disorder for long term were commonly reported.

*“Citalopram for manic-depressive psychosis and trazodone for anxiety disorders are the topmost drugs I use as off-label…”*

P-2

*“I commonly used alprazolam for treating insomnia long term…”*

P-13

##### Subthemes 2: Inform Patient before Prescribing Off-Label Drugs

On questioning the respondents about their practice of informing patients before prescribing off-label drugs; it was observed that the majority of them did not inform their patients.

*“No… Patients do not ask questions about medications most of the times. And even if this happens, No… we don’t give information about off label medication.”*

P-10

*“Not directly as patients are usually not literate enough, but we tell them we are trying something different.”*

P-4

#### 3.2.5. Theme 5: Rationale of Prescribing Off-Label Drugs

The respondents depicted that the appropriate approach to OLDP can be subject to whether the practice is new or old, the urgency of the patient’s condition, and the accessibility of alternative treatment methods.

##### Subthemes 1: They Permit Innovation in Clinical Practice

*“I consider that off-label drugs allows for clinical practice innovation, especially when authorized therapies have failed.”*

P-3

##### Subthemes 2: Earlier Access to Potentially Valuable Medications

*“It provides both patients and clinicians with earlier access to potentially beneficial drugs and enables us to adopt new practices based on emerging data.”*

P-1

##### Subthemes 3: There Is often Crossover in Symptoms from Disease State to Disease State

The respondents opined that most off-label medication has specific therapeutic aims, and in some practice areas, off-label prescribing is both frequent and necessary. They also acknowledged unintentionally prescribing off-label medicines.

*“Sometime the symptoms are overlapping and we do not know that the medicines that we most often administer in our daily practice are considered off-label.”*

P-14

*“Mental health issues are presumably a result of the complicated imbalance of neurotransmitters in different disease stages, and there is often a crossover in symptoms from a disease state to a disease state. It might create several diagnostic problems leading to the use of the drug in a disease that is sometimes off-label.”*

P-5

##### Subthemes 4: The Benefits Associated Outweigh the Associated Risk

The respondents stated that OLDP can potentially have a variety of goals. Most of the respondents opined that for critically ill individuals, off-label usage may be the sole therapeutic and ethically acceptable choice. Patients require access to useful off-label therapies, but they also require protections against unsafe and futile interventions.

*“Sometimes we want the freedom to prescribe medications that are best suited to specific patient requirements, regardless of label.”*

P-9

*“Often there are situations in which we have to evaluate the risks and advantages and offer the best treatment possible to the patients.”*

P-3

*“I believe that if there is reasonable evidence that the benefit of off-label usage outweighs the hazards, that not treating the disease has even higher risks than the off-label prescription, and that there is no suitable alternative therapy, then off-label prescribing can benefit patients…”*

P-11

#### 3.2.6. Theme 6: Suggestions for Reducing the Use of Off-Label Drugs

The respondents were questioned about their suggestions for reducing the OLDP. Four respondents put emphasis on the need of developing more appropriate formulations for psychiatric patients. The respondents focused on increasing the number of clinical trials in psychiatric patients. One respondent suggested training be conducted that specifically targeted the OLDP.

*“The workshops and seminars should be conducted for physicians that give the emphasis on the ethical standards of rational drug prescribing…”*

P-6

##### Subthemes 1: Increasing the Number of Clinical Trials in Psychiatric Patients

The majority of respondents agreed that clinical trials on this vulnerable group of patients are unlikely and this lack of controlled clinical studies in mental patients is the primary reason for OLDP’s widespread use.

*“The lack of clinical trials in psychiatric patients and no interest of drug industries are the major reasons for increased off-label use, so I think these factors should be seen…”*

P-9

##### Subthemes 2: Formulating More Appropriate Drugs for Psychiatric Patients

The respondents believed that the current pace of innovation in psychotropic drugs would play a more significant role in therapeutic management of patients.

*“More suitable formulations made available is the best way to reduce off-label prescription.”*

P-12

*“To reduce such prescription tendencies, more evidence-based formulations for patients are needed.”*

P-3

## 4. Discussion

As far as we possibly know, this is the first qualitative study conducted in a Pakistani setting on psychiatrists’ knowledge, attitudes, and practices related to the use of OLDP. Pakistan is the fifth most populous country in the world, with an ever-increasing need for healthcare resources [29]. Drug treatment is a critical part of clinical practice in patients of all ages, across several diagnostic groups, and in a wide range of settings [30]. Despite the accessibility of numerous classes of psychotropic drugs, a significant number of patients continue to experience distressing symptoms even after a series of licensed pharmacological treatments [31].

The current study discovered gaps in respondents’ knowledge, even though they were well informed about the definition of OLDP. Most of the respondents defined OLDP as utilizing medicines for unapproved indications. However OLDP also include changes in dosage, mode of administration, lack of effectiveness and safety data in a specific age group, lack of dosing information in reference sources, and contraindication of medication usage in a specific age group [32]. This study’s findings validated the necessity for the development and distribution of a standard definition for off-label prescribing to foster a better understanding of this practice. In the current study, the majority of the respondents deemed that OLDP is very common in psychiatry. Psychotropic medications are often given for unapproved purposes in persons suffering from mental health issues and behavioral challenges. Common indicators include sleep difficulties, increased alertness, and self-injurious behavior, and issues associated with the behavioral changes caused by epileptic syndromes and dementia [33]. In the present study the off-label medicines are prescribed by the physicians due to the perceived advantages, as proven in earlier research [34]. The lack of satisfaction with current off-label medication therapy has created a desire for new discoveries, leading to timely adoption of such findings into the medical profession. The most common category for OLDP in the current study was an inappropriate indication. Similar findings were observed in other studies [2,35]. Contrary to this, another study reported dose as the most common reason for OLDP [36]. The OLDP patterns in our study revealed a higher off-label use of benzodiazepines which is not parallel to other studies [2,37]. This might be due to a difference in morbidity pattern, as in our setting, the patients are commonly presenting with depression and schizophrenia, for which psychiatrists prescribed longer use of benzodiazepines to treat insomnia and decrease aggressive symptoms [38]. 

In the current study, some of the respondents were not well-informed about OLDP policies and guidelines. The pharmacy and therapeutic (P&T) committee in the hospital should be seen as the arbitrator of institutional policy addressing off-label pharmaceutical usage, and its judgments should be guided by scientific evidence. When contemplating off-label usage, supporting safety and evidence must be carefully examined, and a risk–benefit analysis must be performed, especially when alternatives with FDA-approved labeling are available. Before approving off-label medication therapy, a systematic method should be implemented to evaluate scientific evidence [31]. Several issues were expressed about OLDP in this study, with the primary worry being that physicians may be prescribing off-label medicines out of habit. They do not consider some of the medicines they regularly give in their daily practice to be off-label. It is worth noting that the majority of respondents expressed concerns about the safety and efficacy of OLDP and believed that improper usage of OLDP increased the chance of ADRs as compared to approved medications. Similar outcomes were reported in earlier research in which the respondents stated their concern about the safety of off-label medicines [39]. This might be explained by the scarcity of patient safety data in pharmacological reference sources, as well as the fact that when medicines are taken off-label, side effects are more common, severe, and underreported [40]. Inappropriate OLDP could generate patient safety concerns as the degree of risk such as side effects, interactions, and adverse events naturally varies depending on the exact off-label use in each case [4].

On questioning the respondents about their practice of informing patients before OLDP, it was observed that the majority of them did not inform their patients. Physicians are required by law to inform patients about potential hazards associated with prescribed medications, since off-label usage should be seen as a risk to the patient [2]. As a result, physicians must adhere to legal requirements that require them to “obtain informed permission from a person before performing a test or providing a therapy—particularly a treatment involving some uncertainty” [41]. The physicians should be obligated to tell patients about off-label usage, but this does not appear to be the case in the current study. In Pakistan, there is no regulatory obligation to inform the patient when a drug prescription is OLDP, but administering drugs outside of the guidelines of the marketing authorization modifies most likely raises professional responsibility [36]. When prescribing outside of these guidelines, the doctor must be able to defend the action with credible professional judgment. The increased emphasis on risk management and evidence-based practice may prompt some trusts to implement systems and processes for monitoring and even guiding the use of psychotropic medications [34].

The respondents were inquired about their suggestions for reducing OLDP. They put emphasis on the need to develop more appropriate formulations and increase the number of clinical trials in psychiatric patients. Failure to research this specific facet of the population exposes patients to risks from medicines and doses that provide different hazards to different groups. For example, there is an emerging understanding that using selective serotonin reuptake inhibitors as off-label for mental problems in young people may raise the risk of depression and suicidal ideation [34]. According to the FDA, physicians can prescribe off-label or unlicensed medicines if they are aware of the benefits of doing so in certain exceptional circumstances [41]. Controlled psychiatric clinical trials are essential for medications to establish the most suitable indications for a specific drug to make sure that patients are not exposed to needless medication hazards [42]. There is a need to enhance psychiatrists’ familiarity with the OLDP problem to improve medication prescribing. The strategies could be implemented to enhance patient access to the most effective treatment, maintain patient safety, and decrease financial risk to the patient by establishing an off-label drug usage policy that incorporates the best practice standards.

The strength of the study is the qualitative study design which assists in gathering the detailed insight of the psychiatrists towards the study objectives. One likely limitation of the present study is that the respondents were interviewed in the hospital settings of Karachi only and therefore transferability cannot be expected. The willingness of the respondents to give answers that are socially acceptable and desirable is also one of the limitations. Moreover, the number of respondents was lower. Further studies are needed to investigate the situation on such practices at a national level.

## 5. Conclusions

The present findings revealed that off-label drug prescribing is common in current medical practice in Pakistan. The respondents have an extensive understanding of off-label usage, yet they are unsure of their conduct when they use medications off label. They were concerned about decreasing such practices by involving in collective decision-making procedures, and were inclined to accept initiatives aimed at ensuring drug safety in patients. The guidelines should be developed at the national level to direct rational medication usage, especially if the potential benefits and risks are complex and time consuming to assess.

## 6. Impact on Practice

- This study is the first of its kind to explore the awareness and practices regarding OLDP in psychiatric practice.
- The study emphasized that extensive training of health care professionals on off-label issues—for instance, the collective utilization of contemporary sources of information, coupled with informal specialized networks—is desired.
- There is a need for improved dissemination of evidence for off-label indications among healthcare teams.
- It ought to be pursued that health experts should inform patients regarding OLDP, however this does not appear to be the situation in current practice.

## Figures and Tables

**Table 1:** Data analysis process.

Phase of Data Analysis	Tasks Accomplished	Member Involved
Phase 1: Familiarization of Data	Transcription, reading, and interpretation of interview transcripts	HR, KJ, and SN
Phase 2: Generation of initial codes	Preliminary, open coding of complete data set	SN, KJ, and HR
Phase 3: Exploration for themes	Alignment of codes into possible themes	SN and HR
Phase 4: Analysis of themes	Confirming themes—assuring the internal homogeneity and external heterogeneity of themes	SS and SN discussed with MA and SJ
Phase 5: Outlining themes	Further modification of themes	SS confirmed with MA and SJ
Phase 6: Finalization of report	Writing the manuscript, selection of explanatory quotes	SS, reviewed by and discussed with SJ and SN

**Table 2:** Demographic characteristics of the respondents.

Characteristics	Frequency
Gender	
Male	8
Females	6
Age (Years)	
30–40	5
40–50	7
>50	2
Working organization	
Private hospitals	9
Public hospitals	5
Experience (Years)	
1–5	7
6–10	4
>10	3
Practice area	
Primary patient care	3
Secondary patient care	5
Tertiary patient care	6

**Table 3:** Description of themes and subthemes.

Themes and Subthemes
Knowledge and concepts about the off-label drugs
Subthemes 1: Kowledge about the definition
Subthemes 2: Knowledge about the examples of widely practiced OLDP
Subthemes 3: Knowledge about the policies and guidelines
Positive attitude
Subthemes 1: Optimistic approach about the safety, efficacy, and quality
Subthemes 2: Willingness to prescribe off-label drugs
Negative attitude
Subthemes 1: Likelihood of ADR
Subthemes 2: Legal vulnerability when using off-label drugs
Current practice of prescribing off-label drugs
Subthemes 1: Category of off-label drug most commonly use
Subthemes 2: Inform patient before prescribing off-label drugs
Rationale of prescribing off-label drugs
Subthemes 1: They permit innovation in clinical practice
Subthemes 2: Earlier access to potentially valuable medications
Subthemes 3: There is often crossover in symptoms from disease state to disease state
Subthemes 4: Benefits associated outweigh the associated risk
Suggestions for reducing the use of off-label drugs
Subthemes 1: Increasing the number of clinical trials in psychiatric patients
Subthemes 2: Formulating more appropriate drugs for psychiatric patients

## Data Availability

The anonymized datasets used and analyzed during the current study are available from the corresponding author on reasonable request.
